# Sternal swelling presenting as tuberculosis: a case report

**DOI:** 10.1186/s13256-021-03008-9

**Published:** 2021-12-06

**Authors:** John Rajan, Khaled Bizanti

**Affiliations:** Department of Paediatric Emergency Medicine, West Middlesex University Hospital, Chelsea and Westminster NHS Foundation Trust, London, UK

**Keywords:** Sternal swelling, Tuberculosis, Pediatrics, Public Health

## Abstract

**Background:**

Tuberculosis continues to be a worldwide public health problem. Despite the noted gradual decline in tuberculosis case rates in the UK, clinicians should still be aware of these unusual presentations. Sternal tuberculosis is an uncommon form of extrapulmonary tuberculosis and it can initially be a diagnostic challenge for paediatricians. These lesions can present with nonspecific signs and symptoms that may mimic malignancy.

**Case presentation:**

We present a case of a 3-year-old African descent girl with a sternal swelling that was confirmed to be *Mycobacterium tuberculosis* complex DNA on gastric aspirate. The child had additional radiological investigations that corresponded accordingly. She was started on quadruple antituberculosis therapy with good outcome.

**Conclusion:**

Tuberculosis sternal abscess is as rare finding, especially in developed countries where tuberculosis is not endemic. Tuberculosis may not always present with pulmonary symptoms in children. There should be a high suspicion of tuberculosis, especially in immigrant population presenting with unusual presentations. Our aim is to increase awareness around atypical presentations of tuberculosis in children. Although, tuberculosis is endemic to underdeveloped countries, clinicians should still be aware of presentations in view of current global migration.

## Background

Tuberculosis presenting as sternal swellings is an unusual presentation in the paediatric population. The few confirmed cases in literature have been from underdeveloped countries where tuberculosis is common. A recent report from Public Health England showed that 4655 people in 2018 were diagnosed with tuberculosis at a rate of 8.3 per 100,000 population. This also happens to be the lowest yet recorded in the UK and a drop of approximately 44% since 2011 (8280 people). However, people born outside the UK accounted for 72% of tuberculosis notifications in 2018 and were also 14 times higher than among those born in the UK [1]. This case illustrates the unusual presentation of a child with a sternal swelling to an acute NHS hospital in West London. Sternal swellings can lead to multiple diagnostic differentials in paediatric patients. A combination of history, examination and an extensive panel of investigations revealed a diagnosis of tuberculosis. The child was started on quadruple antituberculosis therapy with good response.

## Case presentation

**Fig. 1:**
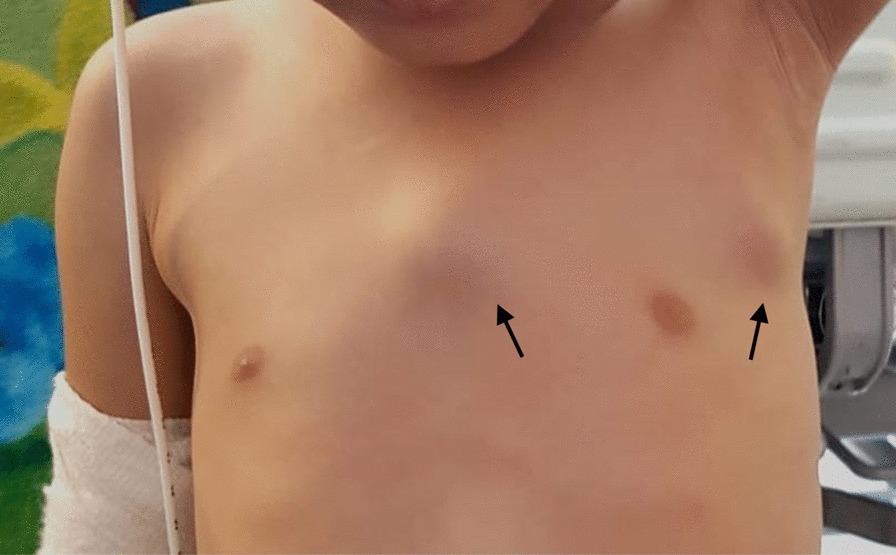
Anterior view of the sternal swelling and the left-sided chest swelling (shown by arrows)

A 3-year-old British African girl of nonconsanguineous parents presented to her local hospital with 4 weeks history of a firm, nontender, enlarging swelling arising over the sternum. A few weeks later, there was an additional swelling noted in her left side of her chest (Fig. 1). This was preceded by 2 months history of manifesting constitutional symptoms of weight loss, lethargy and night sweats. There was no history of fever, cough, breathing difficulties, rash or any local trauma noted. She had been otherwise well prior to this, with no previous medical history or hospital admission. She was on Movicol sachets for constipation management and no other medications. She was born in Kenya and moved to the UK at the age of 1 year. Her immunisations were commenced in Kenya and completed on arrival to the UK as per the national UK schedule. She had received the BCG immunisation, though the scar was not easily visible; her mother confirmed that she had the immunisation. There were no significant associated sick contacts or TB contacts of note. The child’s mother had suffered from extrapulmonary TB approximately 18 years ago and required treatment. She had unfortunately developed side effects from the treatment, probably drug-induced hepatitis as suggested by the jaundice, which led to her treatment being interrupted, though she subsequently completed treatment. She has been well since then and asymptomatic.

On initial examination, the child appeared well nourished and comfortable on room air. She had no signs of respiratory related problems. There was 2 × 3 cm firm, nontender and nonmobile mass over the middle part of her sternum. She also had a second 1 × 1 cm firm, nontender and mobile mass on the left side of her chest. The rest of her physical examination was normal.

**Fig. 2:**
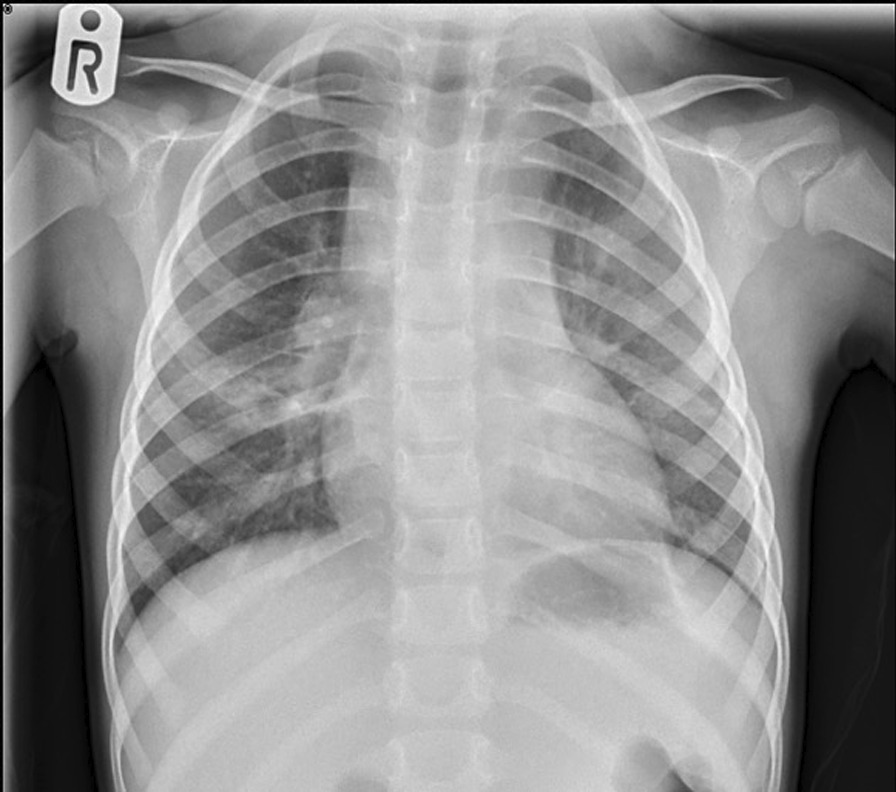
An increased opacification in the right mid-zone and left upper zone

The initial blood tests at the local hospital demonstrated microcytic hypochromic anemia with elevated erythrocyte sedimentation rate (ESR) of 120 mm and C-reactive protein (CRP) of 89. On her initial presentation, she had a chest X-ray, which revealed increased opacifications in the right mid-zone and left upper zone that were thought to be infective in origin (Fig. 2). The patient’s TB Quantiferon Gold test was positive. Her case was discussed further with the paediatric infectious disease team at the tertiary centre. The child had an extensive panel of investigations including repeat baseline bloods and TB Quantiferon test. Blood EBV PCR was performed, which was positive and presumed to be reactivation. CMV PCR and human immunodeficiency virus (HIV)-1/2 antibody analysis were also performed, which were both negative.

**Fig. 3:**
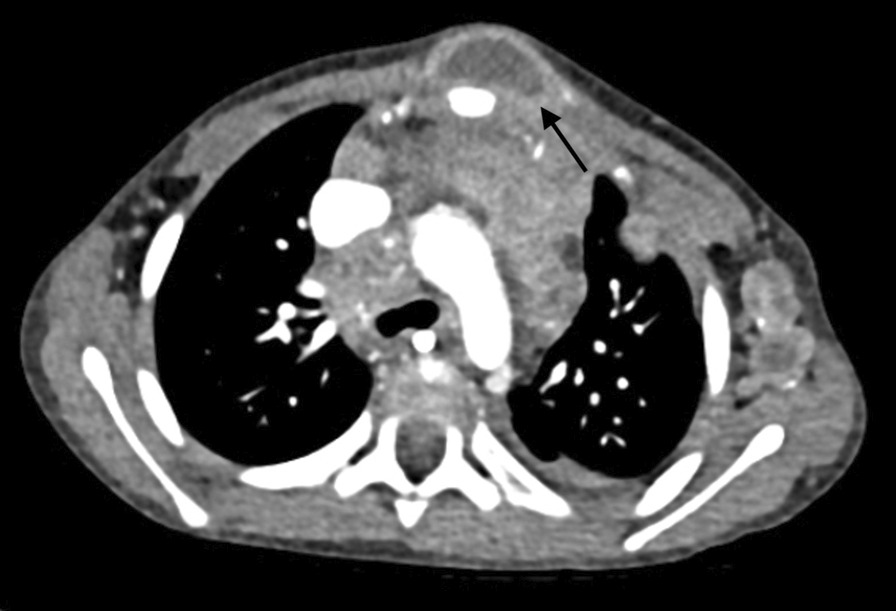
Computed tomography image demonstrating the presternal low-density mass with rim enhancement suggestive of an abscess (shown by arrow)

She underwent an ultrasound-guided biopsy of both lumps. The biopsy result identified necrotic material, pus and areas of granulomatous inflammation including multinucleate giant cells. She had a CT scan at her local hospital that showed a presternal mass with rim enhancement suggestive of an abscess (Fig. 3).

Ziehl–Neelsen staining was negative, though mycobacterial PCR was positive for *Mycobacterium tuberculosis* (MTB) target. Early secreted antigenic target of 6 kDa (ESAT-6) of MTB was positive, which suggested a mycobacterial and non-BCG infection.

This was followed by further imaging to exclude extrapulmonary involvement including MRI head and spine, which showed no features of TB. She had an ultrasound of the abdomen and pelvis, which showed hypoechoic rounded lymph nodes throughout the abdomen. Further immunophenotyping of PB revealed no detectable aberrant expression or maturation asynchrony on B cell, T cell, or natural killer (NK) cell.

**Fig. 4:**
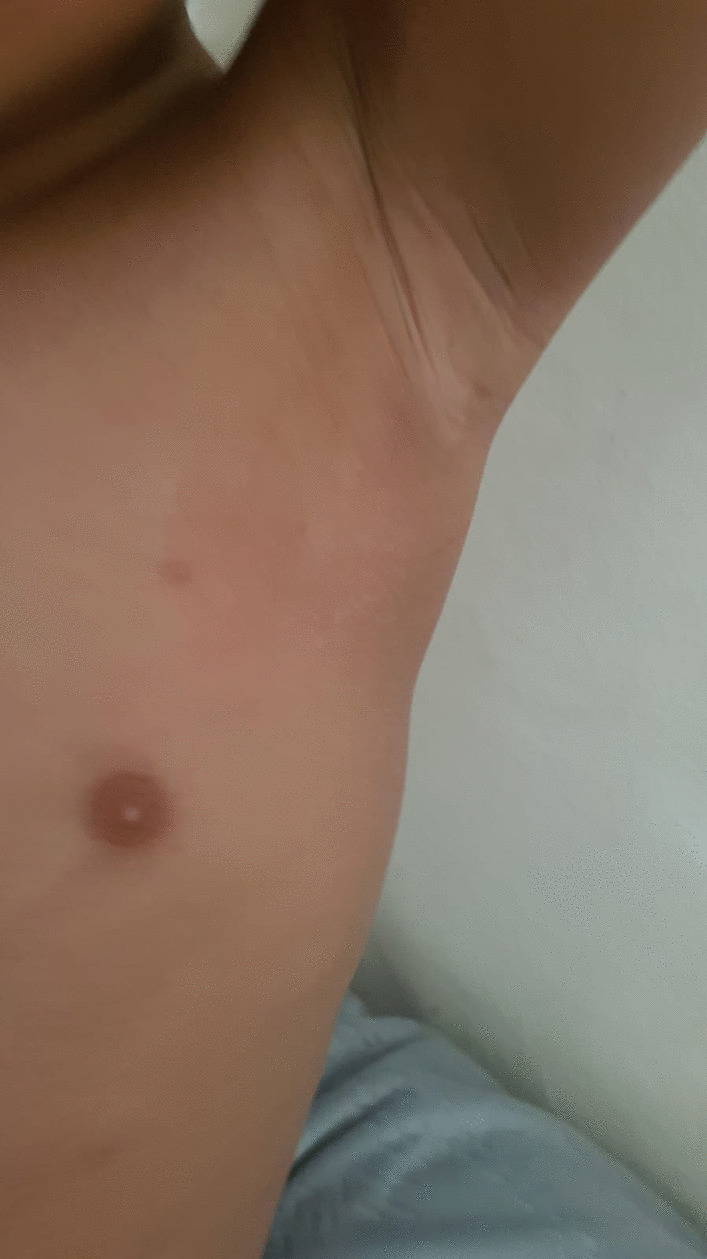
Anterior view illustrating the complete resolution of the left sided chest swelling

**Fig. 5:**
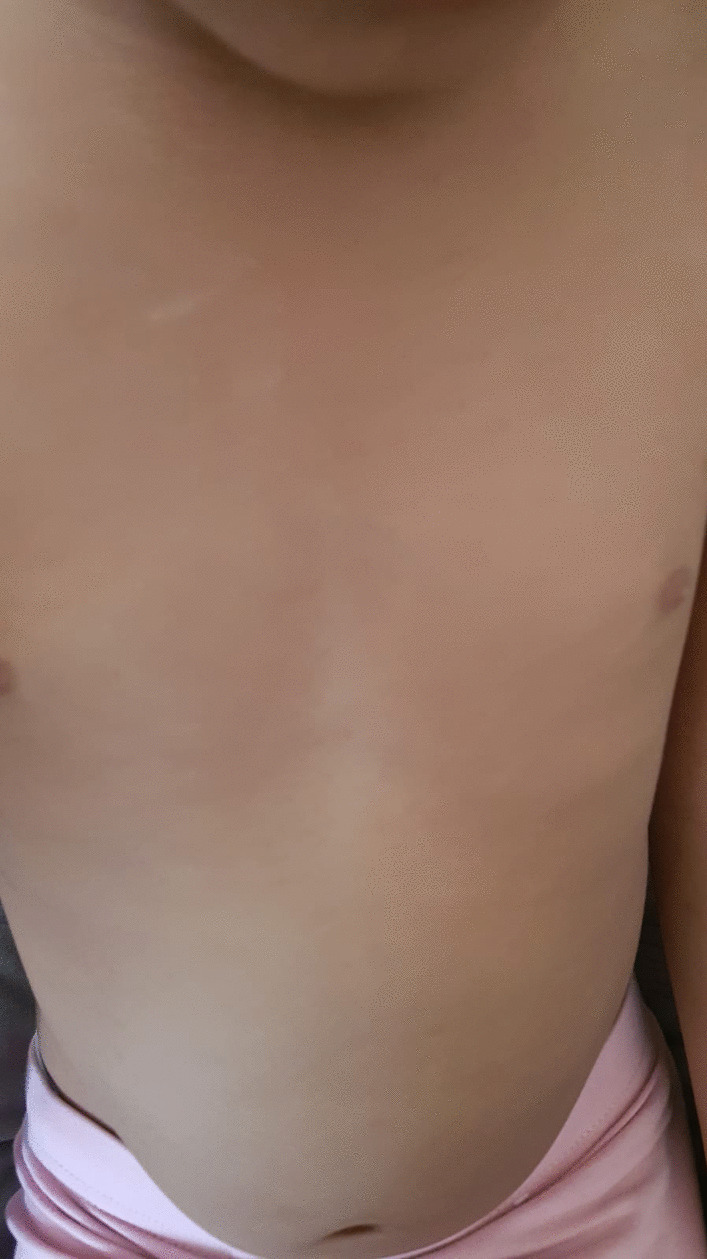
Anterior view illustrating the complete resolution of the sternal swelling

She was commenced on quadruple antitubercular treatment: isoniazid, rifampicin, ethambutol hydrochloride and Zinamide. During her time at the tertiary centre, her inflammatory markers (CRP) peaked at 220 mg/L and she developed fever and required piperacillin/tazobactam, which was later switched to oral co-amoxiclav. She remained clinically well and was subsequently discharged to continue on antituberculosis therapy with further follow-ups scheduled. She is scheduled to have a repeat CT chest in 3–4 months. She has been clinically well and showing good signs of improvement, with the sternal swelling significantly decreasing in size. The treatment has been completed at 6 months with both swellings completely resolved (Figs. 4 and 5).

## Discussion

Tuberculosis is often called the “great imitator,” a common disease with unusual presentations [2]. The incidence of extrapulmonary tuberculosis (EPTB) has been constant as compared with the pulmonary forms [3]. Unfortunately, EPTB can present in various forms and this makes diagnosing these children much more difficult compared with pulmonary TB. Tuberculosis of the sternum is a rare finding, even in countries where TB is prominent [4]. Tuberculosis is no longer an endemic disease of the developing world, as more and more cases are now being reported from developed countries [5]. The increased rate in cases can be attributed to immune-compromised patients (HIV, etc.), large-scale population migration and reactivation TB in the immigrant population.

The diagnosis of these sternal swellings can be difficult as they may present with atypical presentations. Approximately 60–80% of cases of skeletal TB involve the spine or weight-bearing joints, while the sternum is involved in 1% of cases [6–8]. TB of the sternum is a rare form of flat-bone TB that may occur in isolation or in association with pulmonary or lymph node involvement.

In addition, chest wall tuberculosis can mimic bone lymphoma as its development is generally slow, but a history of weight loss, night sweats and itching are usually reported, with an increase in ESR [9]. A high index of suspicion is required for early diagnosis and to initiate prompt treatment to prevent any further complications. Whether surgical intervention is required to treat sternal tuberculosis has yet to be established [10, 11]. Some reports state that the cure rate was approximately 95% with medical therapy alone, while others report that over 25% required concomitant surgical intervention [10]. The 6-month regimen is the same as the medication for pulmonary tuberculosis, but it can be extended to 9 months for sternal tuberculosis because it is difficult to determine the therapeutic response for bone infections [12].

## Conclusion

The diagnosis of sternal tuberculosis is a rare finding even in underdeveloped countries, which can lead to delayed diagnosis. Fortunately, this patient was diagnosed early and appropriate therapy was initiated. A periodic follow-up will be necessary to assess response to treatment and drug resistance, and to monitor possible complications.

## Data Availability

Not applicable.

## Abbreviations

TB: Tuberculosis
EPTB: Extrapulmonary tuberculosis
UK: United Kingdom
NHS: National Health Service
BCG: Bacillus Calmette–Guérin
CT: Computed tomography
EBV: Epstein–Barr virus
CMV: Cytomegalovirus
PCR: Polymerase chain reaction
ESAT: Early secreted antigenic target
PB: Plasmablasts
